# Supplementary material for: Variation in albumin glycation rates in birds suggests resistance to relative hyperglycaemia rather than conformity to the pace of life syndrome hypothesis
Source: eLife. 2025 May 19;13:RP103205. doi: 10.7554/eLife.103205 (PMC12088674; doi:10.7554/eLife.103205)
Supplement: Supplementary file 2. [file elife-103205-supp2.pdf]

Electronic Supplementary Material 2 (ESM2)

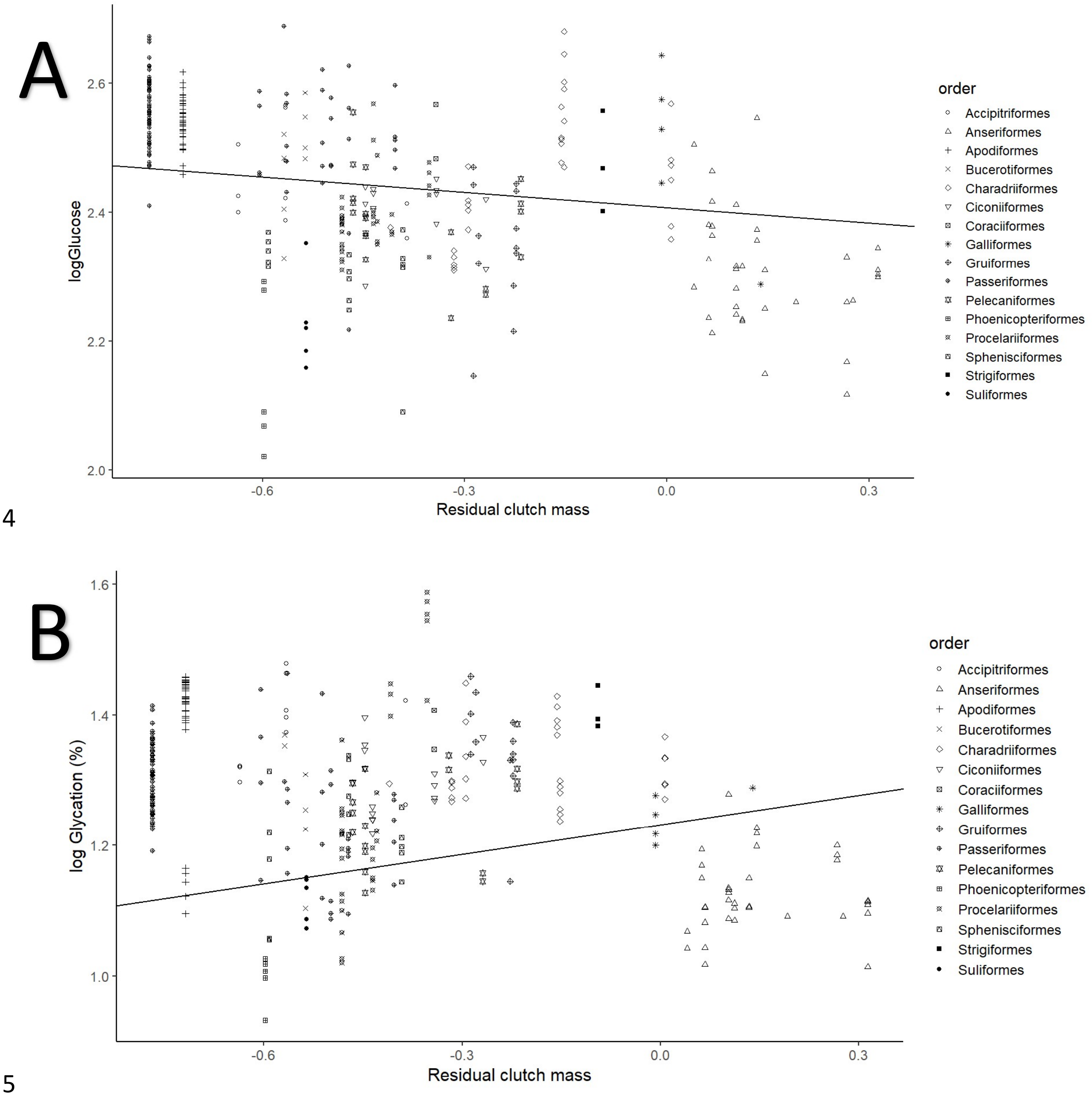

Figure ESM2.1. Variation in **A** log_{10}(plasma glucose) in mg/dl and **B** log_{10}(albumin glycation) levels (%) with respect to residuals of body mass-adjusted clutch mass of the species. This is calculated by multiplying egg mass times clutch size, and then performing a pGLS (see ESM6) of the decimal logarithm of this value against the decimal logarithm of body mass, to finally extract the residuals of such regression. Glycation levels are reported as a percentage of glycated albumin versus total albumin. The legends at right show the different orders to which our species belong.

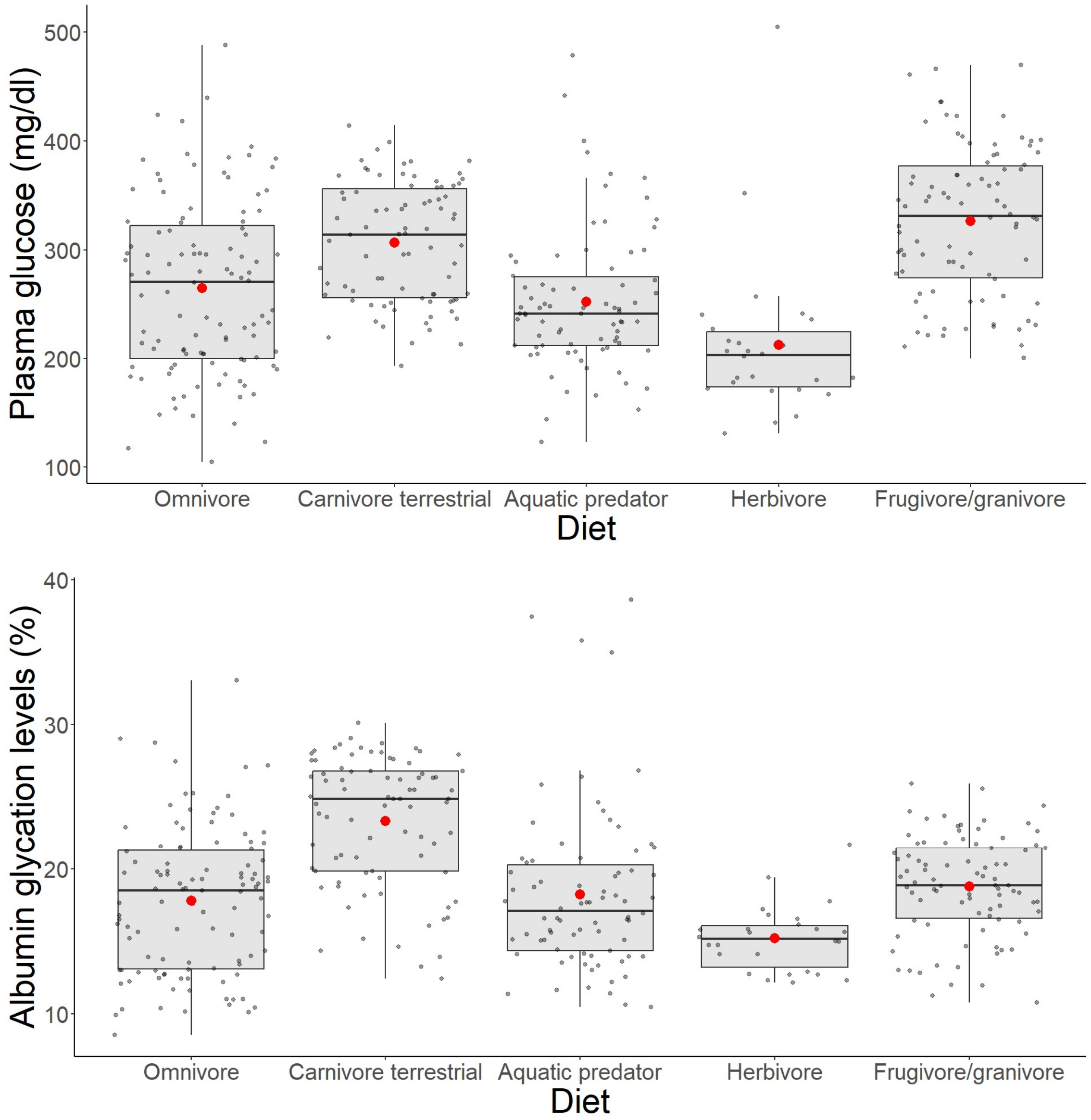

Figure ESM2.2. Representation of raw individual data on (**A**) plasma glucose levels and (**B**) albumin glycation in function of birds' diet. Glucose levels are given in mg/dl, while glycation levels are a percentage of total plasma albumin which is found to be glycated.

A1

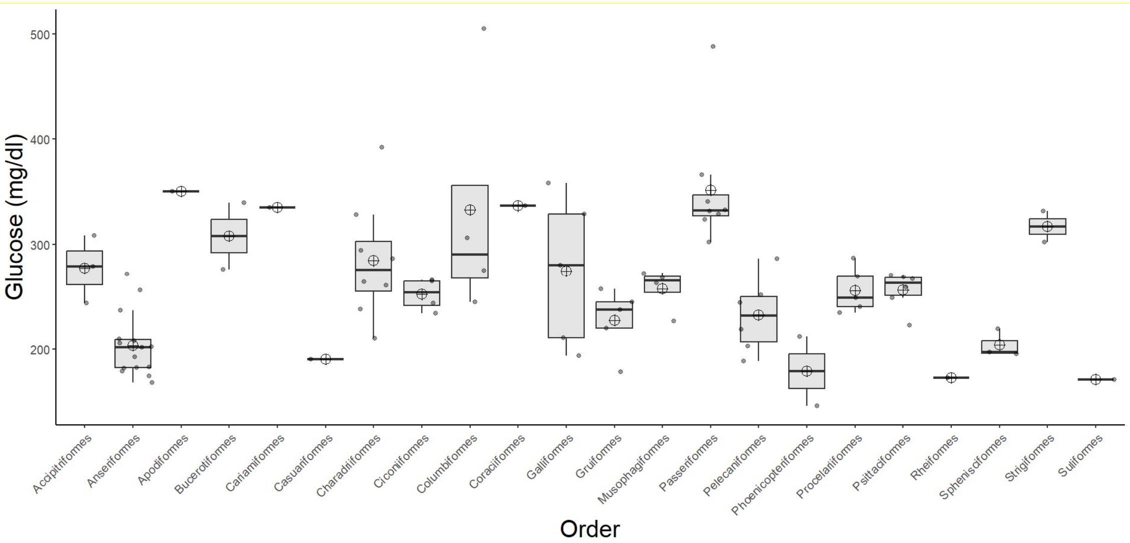
Glucose (mg/dl)
500
400
300
200
Accipitriformes
Anseriformes
Apodiformes
Bucerotiformes
Cariamiformes
Casuariformes
Charadriiformes
Ciconiiformes
Columbiformes
Coraciiformes
Galliformes
Gruiformes
Musophagiformes
Passeriformes
Pelecaniformes
Phoenicopteriformes
Procelariiformes
Psittaciformes
Rheiformes
Sphenisciformes
Strigiformes
Suliformes
Order

A2

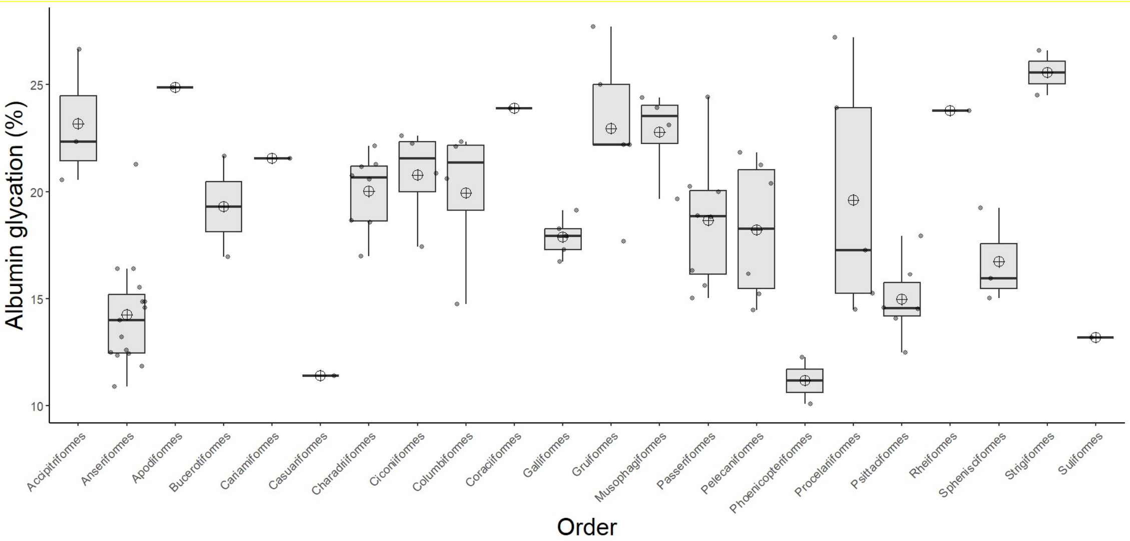
Albumin glycation (%)
25
20
15
10
Accipitriformes
Anseriformes
Apodiformes
Bucerotiformes
Cariamiformes
Casuariformes
Charadriiformes
Ciconiiformes
Columbiformes
Coraciiformes
Galliformes
Gruiformes
Musophagiformes
Passeriformes
Pelecaniformes
Phoenicopteriformes
Procelariiformes
Psittaciformes
Rheiformes
Sphenisciformes
Strigiformes
Suliformes
Order

B1

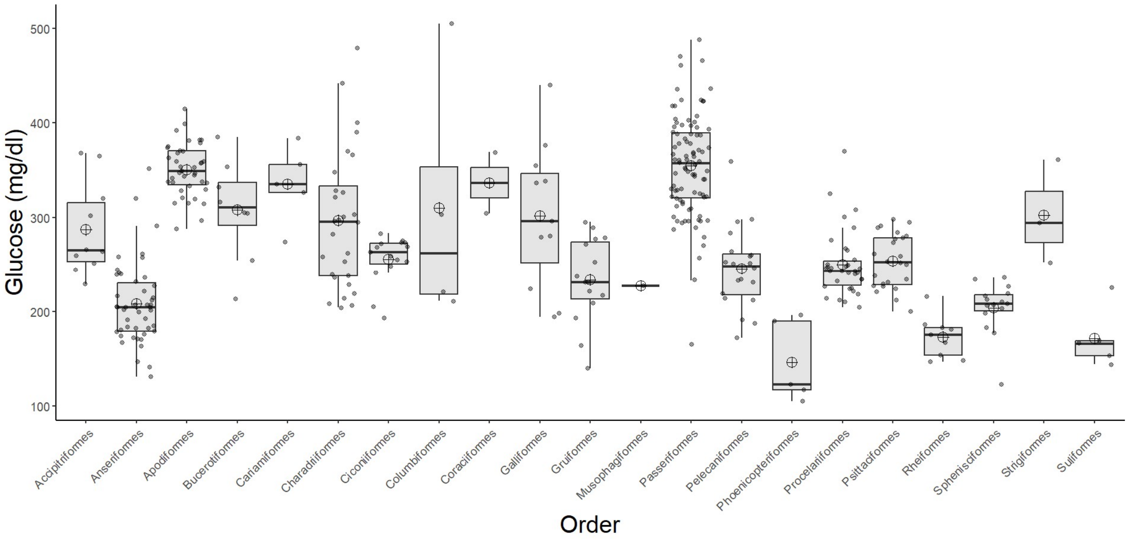
Glucose (mg/dl)
500
400
300
200
100
Accipitriformes
Anseriformes
Apodiformes
Bucerotiformes
Cariamiformes
Charadriiformes
Ciconiiformes
Columbiformes
Coraciiformes
Galliformes
Gruiformes
Musophagiformes
Passeriformes
Pelecaniformes
Phoenicopteriformes
Procelariiformes
Psittaciformes
Rheiformes
Sphenisciformes
Strigiformes
Suliformes
Order

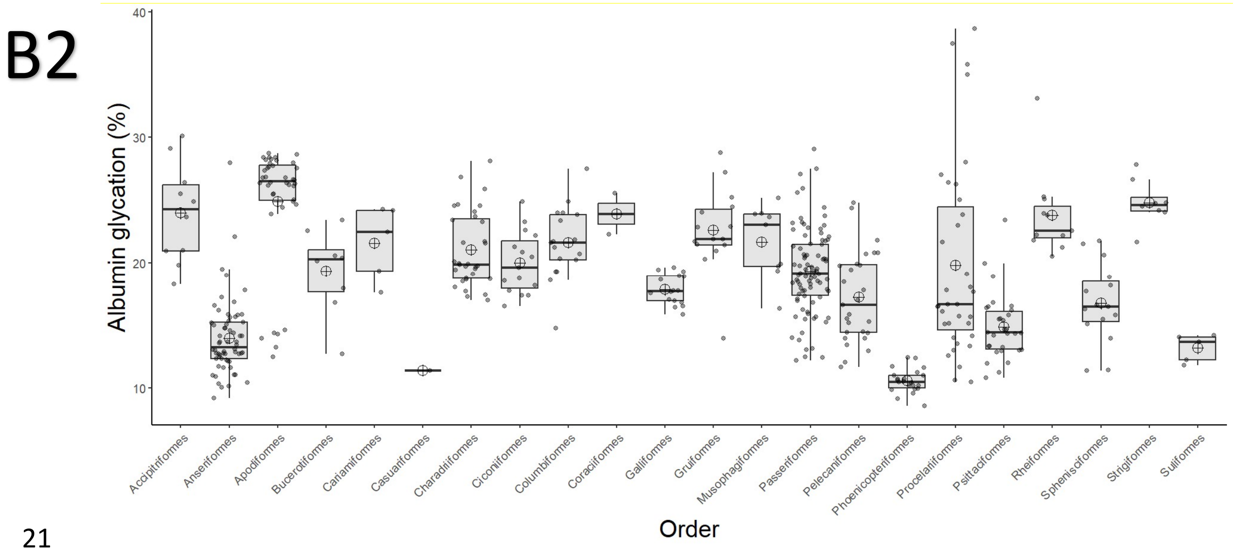

Figure ESM2.3. Variation in (A1-B1) plasma glucose levels in mg/dl and (A2-B2) albumin glycation rates across birds' orders included in our study. Panel A represents average species values and B includes individual values.
